# Assessing Genetic Diversity to Breed Competitive Biofortified Wheat With Enhanced Grain Zn and Fe Concentrations

**DOI:** 10.3389/fpls.2018.01971

**Published:** 2019-01-10

**Authors:** Govindan Velu, Leonardo Crespo Herrera, Carlos Guzman, Julio Huerta, Thomas Payne, Ravi P. Singh

**Affiliations:** ^1^Global Wheat Program, International Maize and Wheat Improvement Center (CIMMYT), Texcoco, Mexico; ^2^Campo Experimental Valle de Mexico, National Institute for Forestry, Agriculture and Livestock Research (INIFAP), Chapingo, Mexico

**Keywords:** biofortification, iron, rye, translocation, wild relatives, wheat, zinc

## Abstract

Breeding wheat with enhanced levels of grain zinc (Zn) and iron (Fe) is a cost-effective, sustainable solution to malnutrition problems. Modern wheat varieties have limited variation in grain Zn and Fe, but large-scale screening has identified high levels of Zn and Fe in wild relatives and progenitors of cultivated wheat. The most promising sources of high Zn and Fe are einkorn (*Triticum monococcum*), wild emmer (*T. dicoccoides*), diploid progenitors of hexaploid wheat (such as *Aegilops tauschii), T. spelta, T. polonicum*, and landraces of *T. aestivum*. This study evaluate the effects of translocations from rye and different *Aegilops* species in a “Pavon-76” wheat genetic background and utilized in the wheat biofortification breeding program at CIMMYT that uses diverse genetic resources, including landraces, recreated synthetic hexaploids, *T. spelta* and pre-breeding lines. Four translocations were identified that resulted significantly higher Zn content in “Pavon 76” genetic background than the check varieties, and they had increased levels of grain Fe as well-compared to “Pavon 76.” These lines were also included in the breeding program aimed to develop advanced high Zn breeding lines. Advanced lines derived from diverse crosses were screened under Zn-enriched soil conditions in Mexico during the 2017 and 2018 seasons. The Zn content of the grain was ranging from 35 to 69 mg/kg during 2017 and 38 to 72 mg/kg during 2018. Meanwhile grain Fe ranged from 30 to 43 mg/kg during 2017 and 32 to 52 mg/kg during 2018. A highly significant positive correlation was found between Zn and Fe (*r* = 0.54; *P* < 0.001) content of the breeding lines, therefore it was possible to breed for both properties in parallel. Yield testing of the advanced lines showed that 15% (2017) and 24% (2018) of the lines achieved 95–110% yield potential of the commercial checks and also had 12 mg/kg advantage in the Zn content suggesting that greater genetic gains and farmer-preferred wheat varieties were developed and deployed. A decade of research and breeding efforts led to the selection of “best-bet” breeding lines and the release of eight biofortified wheat varieties in target regions of South Asia and in Mexico.

## Introduction

Micronutrient deficiency, also known as hidden hunger, is one of the most important challenges facing humanity today. It is caused by a lack of essential vitamins and minerals (primarily vitamin A, iron, and zinc) in the diet and currently affects more than two billion people worldwide (White and Broadley, 2009; WHO, 2017). Pregnant women and young children are particularly prone to acute micronutrient deficiency, which can impair the physical and mental development of children under 5 years of age (Black et al., 2013). Globally, undernutrition contributes to 45 percent of child deaths each year (WHO, 2017), while in low- and middle-income countries it also causes gross domestic product losses of up to 8 percent. Biofortification offers a sustainable solution to increase food and nutritional security for millions of resource-poor consumers where major staples provide most of their dietary energy (Bouis et al., 2011). The wheat (*Triticum aestivum* L.) biofortification program at CIMMYT leading the partnership based global effort to breed competitive wheat varieties with 40% higher Zn concentration (+ 12 mg/kg) over the commercial varieties in the target regions of South Asia (Velu et al., 2011; Singh and Velu, 2017). The primary target nutrient for wheat is zinc (Zn), as millions of resource-poor wheat consumers in South Asia and Africa are prone to Zn deficiency (Stein, 2010).

Wheat is a major staple crop that provides more than 20% of dietary energy and protein consumption worldwide (Braun et al., 2010). Varieties with improved nutritional quality, protein content, high grain yield, and desirable processing quality in adapted elite genetic backgrounds with tolerance to stresses and diseases can help alleviate nutrient deficiencies. Breeding biofortified wheat with enhanced micronutrient concentrations has emerged as a long-term, sustainable solution for micronutrient deficiency (Pfeiffer and McClafferty, 2007). In combination with other strategies, such as supplementation or fortification, biofortification aims to reach micronutrient-deficient rural people who have limited access to formal markets and health care systems. To assure nutritional and food security, it is therefore paramount that suitable biofortified wheat varieties are developed, released, and disseminated for widespread adoption. Since grain nutrition is a non-visible trait, it is essential that new cultivars are not only rich in grain Zn, but that they have a higher yield than current cultivars. This will reduce poverty due to increased incomes and decrease childhood stunting and malnutrition.

Bread wheat (*Triticum aestivum*) is derived from a fertile hexaploid hybrid cross between wild emmer (*T. dicoccoides)* and goat grass (*Ae. tauschii)*. While bread wheat may have evolved several times, it is quite unlikely that its genetic variation is a representative sample of the genetic variation in its progenitors. Recent trait discoveries *in Ae. tauschii* have identified agronomically useful traits that may not be present in bread wheat (Mondal et al., 2016; Vikram et al., 2016). It is recommended that considerable emphasis be placed on exploiting the three species that contributed the wheat A, B, and D genomes due to their long evolutionary history and adaptation to diverse environmental conditions for stress tolerance and potential diversity for nutritional quality traits (Dubcovsky et al., 1998).

The substantial genetic diversity in primary, secondary, and tertiary wheat gene pools serve as raw material for the development of nutritious wheat varieties through breeding (Graham et al., 1999; Monasterio and Graham, 2000; Velu et al., 2014). However, the range of genetic variation for Zn and Fe is considerably lower in improved materials than in landraces and progenitor species. In the case of Zn, the range of variation, particularly among the unadapted species, is sufficient to have a positive impact on human nutrition. However, evidence suggests that mineral concentration is diluted as yield potential rises, increasing the difficulty of using unadapted mineral-rich sources, such as wild relatives to improve adapted wheat (Ortiz-Monasterio et al., 2007). Significant efforts have therefore been made to screen landraces, which tend to have a better agronomic type than wild relatives. Some landraces look very promising, and some have high grain concentrations of Zn and Fe.

Targeted utilization of alien chromosomes through translocations offers an alternative approach for improving nutritional quality along with essential core traits of high yield, durable disease resistance, and end-use quality for making products, such as leavened bread and flat bread, such as *chapattis*. One example is the 1BL.1RS translocation, in which 1RS chromosome from rye has been widely introduced into wheat, where it has replaced long arm of chromosome 1B. This has introduced a new source of leaf rust, stem rust, yellow rust and powdery mildew resistance present on the 1R chromosome. Approximately 60% of bread wheat material from the International Maize and Wheat Improvement Center (CIMMYT) has had this 1BL.1RS translocation at some stage (Rajaram et al., 2002), however, frequency of lines with this translocations has gone down significantly due to new virulent strains of rust fungus and the negative effect of 1RS translocation on end-use quality. Though the effect of this translocation on grain Zn concentration has not yet been evaluated.

Synthetic hexaploids (using *T. durum* or *T. dicoccum* and diverse sources of *Ae. tauschii*) offer large variability for agronomic and nutritional quality traits. There have been successful introgressions of quantitative traits from synthetic hexaploids into adapted germplasm, a process that involves limited backcross populations, which are then evaluated for agronomic traits and grain Zn and Fe concentrations (Velu et al., 2016). These introgressions have been utilized in released varieties, such as “WB02,” and “Zinc-Shakti,” which have Zn levels 20–40% higher than local varieties (Singh and Velu, 2017). Capturing genetic variation from wild relatives and landraces through targeted crosses and early generation selection for agronomic and disease resistance and later generations for yield and yield stability and Zn concentrations showed large number of lines combine high yield and high Zn.

CIMMYT's biofortification program exploits diverse genetic resources and utilizes new wheat varieties that are high-yielding, more heat and drought tolerant, and have better end-use quality. Thus, superior agronomic traits are inherent in the biofortified wheat varieties, along with nutrition. This study aimed to test the effect of translocations from different rye and *Aegilops* species on grain Zn content in “Pavon 76,” wheat genetic background across 2 years in Mexico (Set I). Advanced lines derived from crosses with diverse progenitors having enhanced Zn and Fe contents were also evaluated and selection was carried out to achieve the dissemination of agronomically superior wheat varieties with significantly increased Zn and Fe concentrations (Set II).

## Materials and Methods

### Experimental Site

Field experiments were conducted from 2016 to 2018 at the Norman E. Borlaug Experimental Station (CENEB) in Ciudad Obregon, Mexico (27°20′N; 109°54′ W; 38 masl). Irrigation was supplied five times during the growing season to avoid water stress.

### Experimental Design and Crop Management

#### Set I

The historic spring bread wheat variety “Pavon 76,” and the 62 translocated lines were sown in a randomized block design with two commercial checks (“Kachu” and “PBW 343”) in 2 years (2017, 2018). “Kachu” is a “Kauz” derived high-yielding variety grown more widely in India; “PBW 343” (Attila) is a historical variety still grown by farmers in South Asia. Each genotype was planted in a double row of 1 m length with a bed to bed distance of 80 cm. All recommended agronomic practices were followed. The commercial form of ZnSO_4_·7H_2_O was applied as basal fertilizer, along with 50% of the recommended 200 kg/ha nitrogen and 100% of the 50 kg/ha phosphorus fertilizers. The remaining 50% or 100 kg/ha N was applied as top dressing during the second irrigation, or 30 days after sowing. At maturity, whole plots were harvested and 30 g grain samples from each plot were used for micronutrient analysis. We also measured thousand kernel weight (TKW).

#### Set II

Advanced lines were derived from diverse crosses of CIMMYT elite breeding lines with high Zn synthetic hexaploids, landraces, and other sources. The simple and top cross (three-way) derivatives were advanced to large F2 populations (>2,000 plants/cross) and F3, F4, F5 bulks of about 400–800 plants were grown and plants resistant to yellow rust and leaf rust were bulked separately in Toluca and at CENEB. From the F4 and F5 grown in Toluca, yellow rust resistance and *Septoria tritici* blotch resistant plants were harvested and individual head-rows were sown. After visual selection of individual heads based on plump and bold grains seed were sown at CENEB as head-rows. Head-rows were evaluated for agronomic characteristics and resistance to leaf and stem rusts compared to repeated checks. The best performing F6 head-rows exhibiting resistance to rusts, and having superior agronomic performance, bold and plump grain types were analyzed for Zn and Fe concentrations. Grain protein content and grain hardness were also measured using a Near Infra-Red Spectroscopy (NIRS) assay at the CIMMYT Wheat Quality Laboratory on advanced selected lines.

F6 lines were advanced to the first yield trials, grown in an alpha-lattice-Latinized design with three replicates both in the 2017 and the 2018 crop seasons. Each trial comprised of two checks and 28 entries in both seasons. Trials were planted in November and harvested in late April and five times surface irrigation (>500 mm) was provided to avoid water stress. For weed control, 20.6% flucarbazone-sodium was applied at the rate of 0.5 L/ha to control narrow leaf weeds and a mix of Starane (fluroxypyr-meptyl, 45.52%) and Buctril (bromoxynil octanoate, 31.7%), was applied at the rate of 0.4 and 0.3 L/ha, respectively for broad leaf weeds just before sowing. For insect control, Admire (imidacloprid, 30.2%) was applied at the rate of 0.75 L/ha during tillering/booting stage of the crop. Approximately 200 kg/ha N was applied. The commercial form of Zn fertilizer (ZnSO_4_·7H_2_O) was applied to optimize and homogenize available soil Zn in order to reduce soil Zn heterogeneity at CENEB.

Whole plots were harvested after physiological maturity and grain yield was measured. Quality traits were analyzed using 50 g seed from each advanced selected wheat lines. TKW, grain Zn, and Fe concentrations scores were measured for all entries.

### Micronutrient Analysis

Grain samples weighing ~30 g and free from dust particles, chaff, glumes, and other plant material were prepared for determining micronutrient concentration and thousand kernel weight. Thousand kernel weight (TKW) was measured with a SeedCount digital imaging system (model SC5000, Next Instruments Pty Ltd), New South Wales, Australia. Grain Zn and Fe concentration (in mg/kg) were measured by a bench-top XRF machine (Oxford instruments, UK) (Paltridge et al., 2012). This Energy-dispersive X-Ray Fluorescence spectrometry (EDXRF) technique have been standardized to perform non-destructive elemental analysis of whole grain wheat samples for Zn and Fe testing at CIMMYT. The EDXRF methods was developed using a large set of randomly selected wheat samples with variable seed properties (30–70 mg/kg Zn and 30–50 mg/kg Fe contents) (Paltridge et al., 2012). As a highly significant positive correlation was observed in a preliminary analysis between the EDXRF and the Inductively Coupled Plasma Atomic Emission Spectrometry (ICP-AES) measurements when used for Zn and Fe (*r* = 0.9 and 0.9, respectively; *P* < 0.001) analysis, with lower than 5% coefficient of variation (CV), the EDXRF found to be a rapid, economical and near accurate measurement of grain Zn and Fe in whole grain wheat samples.

### End Use Quality Analysis

Competitive high yielding, high Zn candidate lines (770 lines from the F6 generation) were analyzed for processing and end-use quality parameters. Dough deformation work (W), and dough strength vs. extensibility (P/L) were measured using Chopin Alveograph (Chopin Technologies, France). To assess the end-use quality of yeast-leavened bread, pup loaves were baked as pan bread using AACC method 10–09 (AACC, 2000) using the (Guzman et al., 2015) method for the adjustment of the optimal water absorption. Bread loaf volume was measured by rapeseed displacement in accordance with AACC method 10–05.01 (AACC, 2000). Wheat kernel hardness was measured based on particle size index measurement according to AACC Method 55–30.

### Statistical Analysis

Statistical analyses was carried out using PROC MIXED in SAS 9.2 (SAS Institute, Cary, NC, USA) software. Mean comparisons between the original line “Pavon 76” and its translocated lines (Set I) were made for all four traits (grain yield, TKW, Zn, Fe) in the study using Tukey's test. Broad-sense heritability (H^2^) was estimated across environments for Zn and Fe content using the breeding lines (Set II), using the formula H^2^ = $\sigma_{\text{g}}^{2}$/($\sigma_{\text{g}}^{2}$ + $\sigma_{\text{gl}}^{2}$/l + $\sigma_{\text{e}}^{2}$/rl), where $\sigma_{\text{g}}^{2}$ is genotypic variance, $\sigma_{\text{gl}}^{2}$ is the genotype × location variance, and $\sigma_{\text{e}}^{2}$ is the residual error variance for r replicates and l locations. Genotypic values (i.e., line means of Set II) were estimated as Best Linear Unbiased Estimators with a random effect for replicates nested within each environment.

## Results

### Identification of High Zn Wheat Lines With Rye Chromosome Translocations

The sixty-two “Pavon 76” translocation lines (Set I), which showed high variation in their morphological properties (heading date and plant height, data not shown) also showed high variation in both Zn and Fe content, but there was also a significant variation in the thousand kernel weight (TKW) of the samples as well (Table 1). Grain Zn content varied from 38.6 to 57.6 mg/kg (mean = 47.1 mg/kg), whereas Fe content ranged from 32.0 to 53.0 mg/kg (mean = 36.6 mg/kg), next to the TKW ranges from 31.5 to 48.0 g (mean = 37.6 g). Tukeys' test showed that four translocation lines had significantly outstanding Zn content (LSD5% = 3.6) with more than 10 mg/kg Zn advantage over trial mean.

**Table 1:** Summary of mean values for Fe and Zn concentration and TKW among Pavon 76 translocation lines (Set I) (2017, 2018).

	**Genotype identifier (GID)**	**Cross**	**Fe (mg/kg)**	**Zn (mg/kg)**	**TKW (g)**
1	7615612	Pavon 76, + 1R(1B)	33.7	47.7	36.5
2	7615614	Pavon 76, + 1R(1A)	34.5	53.0	36.5
3	7615616	Pavon 76, + 1R(1D)	35.9	50.4	36.0
4	7615618	Pavon 76, + 1R.1D5+10-2(1D)	34.4	49.8	34.5
5	7615620	Pavon 76, + 1Rinv(1A)	35.5	47.5	34.5
6	7615622	Pavon 76, + 1R(1D), PAVON7	33.4	49.1	35.0
7	7615624	Pavon 76, + 1Rr(1A), PAVON8	49.1	49.5	35.0
8	7615626	Pavon 76, + 1Rr(1B), PAVON9	32.4	45.2	37.0
9	7615628	Pavon 76,' + 1Rr(1D), PAVON 10	37.8	57.6	34.5
10	7615630	Pavon 76, + 1Ri(1B)	35.7	52.5	36.0
11	7615632	Pavon 76, + MA1S.1RLe(1A)	36.0	51.1	35.0
12	7615634	Pavon 76, + MA1S.1RLe(1B)	33.7	49.0	39.0
13	7615636	Pavon 76, + MA1S.1RLe(1D)	37.3	55.7	39.5
14	7615638	Pavon 76, + 2Rrec(2B)	36.9	48.9	35.5
15	7615640	Pavon 76, + 4Aril	32.7	45.9	39.5
16	7615644	Pavon 76, + 1RS.1AL	35.8	43.2	37.0
17	7615646	Pavon 76, + 1RS.1BLcim	34.3	44.5	38.5
18	7615648	Pavon 76, + 1RS.1BL gnr	35.8	48.1	38.0
19	7615650	Pavon 76, + 1RS.1DLbb	36.3	47.7	42.0
20	7615652	Pavon 76, + 1RS.1DLw	32.1	43.6	37.5
21	7615654	Pavon 76, + 1RS.1ALrh	41.7	42.9	37.5
22	7615656	Pavon 76, + 1RSe.1AL	53.0	44.0	39.5
23	7615658	Pavon 76, + 1RSe.1BL	36.5	43.1	40.0
24	7615660	Pavon 76, + 1RSe.1DL	35.6	43.8	40.5
25	7615662	Pavon 76, + 1RSv.1AL	35.9	44.4	38.5
26	7615664	Pavon 76, + 1RSv.1BL	34.7	46.8	38.5
27	7615666	Pavon 76, + 1RSv.1DL	33.9	44.8	39.0
28	7615668	Pavon 76, + 1RSi.1BL	36.8	47.5	39.5
29	7615670	Pavon 76, + MA1	36.2	47.5	39.0
30	7615672	Pavon 76, + MA2	37.5	51.9	38.5
31	7615674	Pavon 76, + Te1	35.4	47.4	38.0
32	7615676	Pavon 76, + Te2	34.8	46.9	36.5
33	7615678	Pavon 76, + 1RSe.1BLv	35.5	47.0	35.5
34	7615680	Pavon 76, + 1AS.1RLe	35.7	47.8	34.0
35	7615682	Pavon 76, + 1BS.1RLe	34.5	47.7	35.0
36	7615684	Pavon 76, + 1DS.1RLe	44.2	48.1	35.5
37	7615687	Pavon 76, + 1DS.1RLbb	36.2	52.9	35.5
38	7615688	Pavon 76, + 2RS.2BLcs	35.1	49.6	39.0
39	7615690	Pavon 76, + 2BS.2RLcs	36.1	45.3	35.0
40	7615692	Pavon 76, + 2BSp.2RLbl	32.0	44.2	36.0
41	7615694	Pavon 76, + 2D(s) + 2”	36.3	44.1	36.5
42	7615697	Pavon 76, + 2D(s) + 4”	40.5	56.1	32.5
43	7615699	Pavon 76, + 3RS.3DLrh	34.0	45.0	39.5
44	7615700	Pavon 76, + 3RS.3DLcs	32.3	44.4	37.5
45	7615702	Pavon 76, + 3DS.3RLcs	36.7	44.0	38.0
46	7615704	Pavon 76, + 7DS.4RLm	33.6	45.4	37.5
47	7615706	Pavon 76, + 5RS.5ALcs	34.0	42.8	36.5
48	7615708	Pavon 76, + 5RS.5BLe	35.2	43.9	38.0
49	7615710	Pavon 76, + 5D.5R-1”	40.3	44.3	39.0
50	7615712	Pavon 76, + 5RS.5DLrh	32.7	42.3	37.5
51	7615714	Pavon 76, + 6BS.6RLbb	34.9	48.1	41.0
52	7615716	Pavon 76, + 7A.7S-S3	36.7	38.6	40.0
53	7615718	Pavon 76, + 7A.7S-L7	33.8	43.8	41.5
54	7615720	Pavon 76, + 7A.7S-L5	39.3	44.8	42.0
55	7615722	Pavon 76, ' + 7A.7S-Gb5	50.5	43.1	42.0
56	7615728	Pavon 76, 1AS.#2L	34.4	52.1	31.5
57	7615730	Pavon 76, 1RSi.1BL	47.2	44.6	38.5
58	7615732	Pavon 76, 1RS.1AL” 1RS.1DL”	36.3	54.0	43.0
59	7615734	Pavon 76, + 2BS.2RLcs, PAVON85	35.4	43.9	36.5
60	7615736	Pavon 76, + 2R.2B”	35.7	50.4	35.0
61	7615739	Pavon 76, + 1D+9”	33.4	44.7	35.5
62	7615740	Pavon 76, + 2AS.2RLcs	34.8	48.1	32.5
parent	7615724	Pavon 76	32.0	39.5	38.5
check1	2430154	PBW343 (Check)	47.4	48.5	45.0
check2	4755014	Kachu #1 (Check)	36.5	47.5	44.0
		Mean	36.6	47.1	37.6
		Minimum	32.0	38.6	31.5
		Maximum	53.0	57.6	48.0
		SD±	4.4	3.8	2.8
		LSD _5%_	1.6	3.6	2.9
		H^2^	0.52	0.80	0.90

*H^2^, broad sense heritability; LSD, least significant difference; SD, standard deviation; TKW, thousand kernel weight*.

Grain Zn concentration of entry GID: 7615628 disomic line with translocation from 1Rr rye chromosome showed the highest grain Zn with 9.6 mg/kg Zn concentration over the average Zn content of the checks, but had 18.1 mg/kg higher Zn content than the “Pavon 76.” This was followed by entry GID: 7615697 containing chromosome 2Ds from rye with 8.1 mg/kg improvement in Zn content compared to the average of the controls, but have 16.6 mg/kg higher Zn content than “Pavon 76.” Lines GID: 7615636 and 7615732 also had outstanding Zn content having MA1S.1RLe and 1RS.1AL translocations, respectively. These lines were selected to use in breeding programs aiming to increase the mineral content of wheat. Compared to “Pavon 76,” the Fe content of these four lines also increased by 4.3–8.5 mg/kg as a result of translocation, but they had lower Fe content than the check line, “PBW343.”

**Figure 1:**
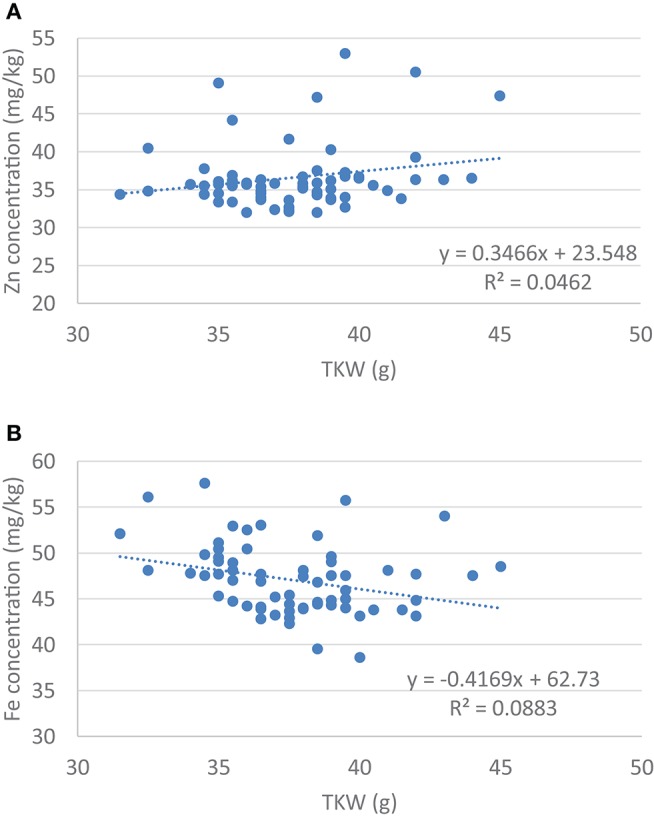
Regression analysis between **(A)** Grain Zn concentration and the thousand kernel weight (TKW) and **(B)** Grain Fe concentration and thousand kernel weight (TKW).

The size and weight of the kernel was expected to influence the Zn and the Fe concentration in the seed, so a correlation analysis was carried out. Data showed that there was a significant negative correlation between the TKW and the Zn content of the kernel (*r*_5%_ = −0.297, *r*_5%krit_ = 0.250, *n* = 62), but no correlation was found in case of the Fe content (*r*_5%_ = 0.215, *r*_5%krit_ = 0.250, *n* = 62). At the same time regression analysis did not show any association between the size of the kernel and its Zn or Fe content (Figure 1). This may refer to the possibility to select lines with stably high Zn and/or Fe content without dilution effects of the seed size. There was no correlation between the Zn and Fe content in case of the wheat/rye translocation lines (*r*_5%_ = 0.043).

### Stability, Heritability of Zn and Fe Concentration

Combined analysis across years for Pavon 76 translocation lines (Set I) showed significant year effects on grain Zn and TKW (*P* < 0.001). Broad sense heritability was high for Zn and TKW (*H*^2^ = 0.80 and 0.90, respectively) while it was intermediate for Fe content (*H*^2^ = 0.52) (Table 1), with a coefficient of variation below 10%, referring to good management of trials across years.

**Figure 2:**
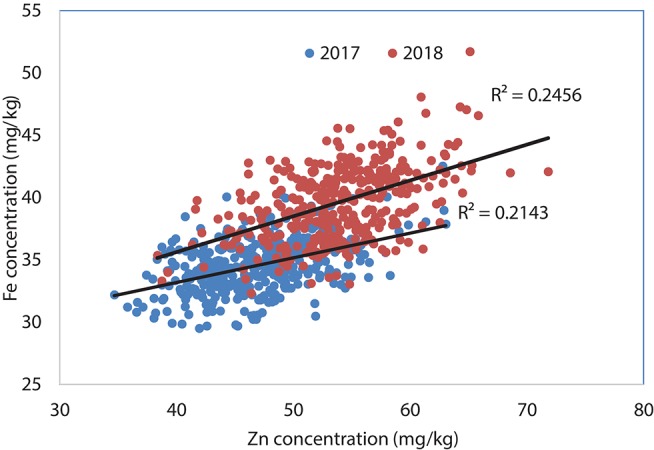
Association between grain Zn and Fe concentrations using selected lines of Set II, 2017 and 2018.

Analysis of Set II lines showed similar results with high heritability values for Zn (0.79 and 0.83 in 2017 and 2018, respectively) and TKW (0.85 in 2017) and medium level for Fe content (0.67 and 0.66 in 2017 and 2018, respectively) (Table 2). However, highly significant positive correlation has been observed between Fe and Zn concentrations during 2017 (*R*^2^ = 0.25; *P* < 0.05) an 2018 (*R*^2^ = 0.21; *P* < 0.05) crop seasons (Figure 2).

**Table 2:** Summary of mean, maximum, and minimum values for Fe and Zn concentration and grain yield among advanced high Zn lines (selection from Set II), 2017 and 2018.

**Statistics**	**Grain Zn (mg/kg)**	**Grain Fe (mg/kg)**	**Grain yield (t/ha)**	**TKW (g)**	**Test weight (kg/hL)**	**Grain protein (%)**	**Grain hardness (PSI)**	**Loaf volume (cm^**3**^)**
**2017**, ***N*** **= 416 Lines**								
Trial mean	52	35	6.2	47.8	79.3	13	71	724
Range	35–69	30–43	5.8–8.1	40.8–61.0	73.8–82.3	11.2–15.5	23–86	500–935
H^2^	0.79	0.67	0.82	0.85	0.75	0.6	0.75	0.7
CV (%)	7.8	9.8	5.1	8	9	8.5	10	12
LSD	4	3.2	0.64	3.3	1.4	0.68	6	67.13
**2018**, ***N*** **= 354 Lines**								
Trial mean	53	40	6.8	50.8	80.1	13.2	63	720
Range	38–72	32–52	4.3–8.4	41.3–59.7	76.4–83.3	11.5–15.3	49–79	555–855
H^2^	0.83	0.66	0.79	0.86	0.71	0.7	0.65	0.74
CV (%)	8.8	10.2	4.61	5.52	8.7	6.8	9.8	10.4
LSD	5.3	3	0.63	4.1	2.2	0.87	5.4	58.7

*CV, coefficient of variation; H^2^, broad sense heritability; LSD, least significant difference; PSI, particle size index; SD, standard deviation; TKW, thousand kernel weight*.

Next to Zn and Fe content, the heritability of other main seed components and properties were also studied. Grain yield and physical properties of the seed were highly heritable with 0.82, 0.79 (2017, 2018) heritability values for grain yield, 0.75 and 0.71 for test weight (2017, 2018), 0.75 and 0.65 for grain hardness (2017, 2018), 0.6 and 0.7 for protein content (2017, 2018), and 0.7 and 0.74 for loaf volume (2017, 2018).

### Breeding for High Zn Wheat Genotypes

**Figure 3:**
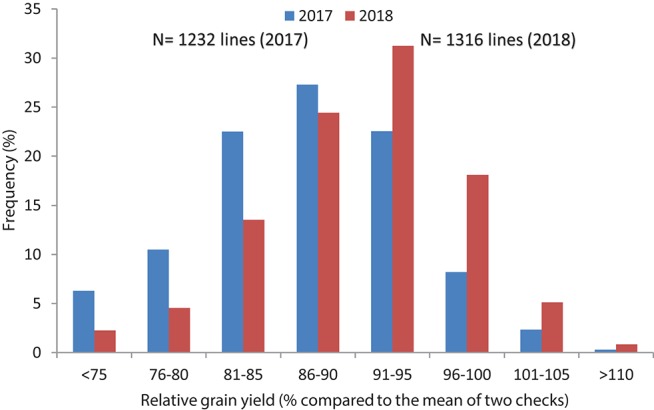
Frequency distribution of relative grain yield potential of high Zn lines compared to checks in yield trials (Set II) of 2017 and 2018.

In Set II experiment, where breeding lines were evaluated, highly significant differences were observed between genotypes during the 2017 and 2018 crop seasons. The average yield potential during the 2017 season was 6.2 t/ha, with a range of 5.8–8.1 t/ha, whereas it was 6.8 t/ha mean with the range of 4.3 to 8.4 t/ha in 2018 (Table 2; Figure 3). About 15 percent of the advanced lines achieved 95–110 percent of the yield potentials of the two check varieties (“Kachu” and “Borlaug,”) whereas in 2018, 24% of the lines showed 95–110% grain yield potential compared to the two checks (which were “Mayil” and “Borlaug 100” in 2018).

**Figure 4:**
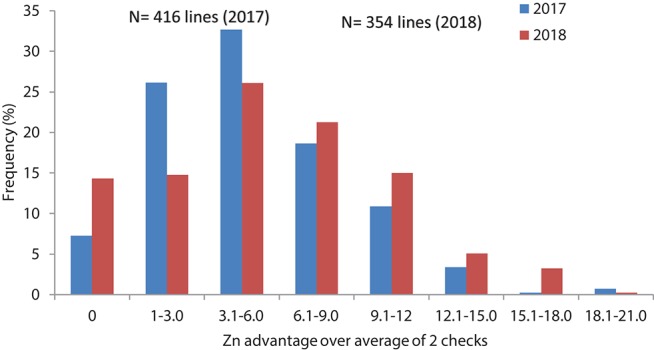
Frequency distribution of grain Zn concentration changes related to check average in advanced high Zn lines (selection from Set II), in 2017 and 2018.

Grain samples yielded similarly or better than checks were analyzed for grain Zn and Fe content with XRF method. Results showed about 4.8 and 5.4 mg/kg average Zn increase in 2017 and 2018, respectively for the selected lines compared to the checks average, while the changes in individual lines could go up to 21–23 mg/kg Zn increase above the checks average (Table 2, Figure 4).

Grain Zn varied from 35 to 69 mg/kg (mean = 52 mg/kg) and 38 to 72 mg/kg (mean = 53 mg/kg) during the 2017 and 2018 seasons, respectively, whereas grain Fe ranged from 30 to 43 mg/kg (mean = 35 mg/kg) during 2017 and 32 to 52 mg/kg (mean = 40 mg/kg) during 2018 (Table 2).

In terms of end use quality most of the lines expressed better processing quality and semi-hard to hard-grain texture, except for six entries that had tenacious gluten property. TKW varied from 40.8 to 61.0 g (mean = 47.8 g) in 2017 and 41.3 to 59.7 g (mean = 50.8 g) during 2018, while test weight ranged from 73.8 to 82.3 kg/hl (mean = 79.3 kg/hl) in 2017 and 76 to 83 (80 kg/hL) during 2018 crop season, indicating potentially good milling quality (Table 2). Meanwhile loaf volume ranged from 500 to 935 cm^3^/100 g of flour (mean = 724 cm^3^) in 2017 and from 555 to 855 cm^3^ (mean of 720 cm^3^) in 2018. The protein content of these lines was also variable changing between appr. 11.5 and 15.5% in both years. These results indicate that high Zn candidate lines could possess desirable end-use and processing qualities for making various types of flat and yeast leavened breads.

About twenty best breeding lines were identified and selected for further testing in different environments as they had 8.3–24 mg/kg advantage in their Zn concentration and 0–6% yield superiority for grain yield compared to the controls (Table 3). These lines had test weight ranging from 78.2 to 82.5 kg/hl, TKW from 44.3 to 57.2 g, PSI form 52 to 14.4, grain protein content from 12.2 to 14.4%, Fe content from 33.1 to 57.2 mg/kg, Alveograph W from 112 to 358 (^*^10^−4^J), Alveograph P/L value from 0.9 to 2.9 and loaf volume from 610 to 780 cm^3^. This means that selected high Zn lines still have variable physical and compositional properties, but their breadmaking quality is expected to be excellent for most lines.

**Table 3:** Characterization of the best genotypes from most recent high Zn wheat lines (2018).

**No**	**GID**	**Cross name**	**Grain yield (t/ha)**	**Grain yield % of Borlaug 100**	**Zn (mg/kg)**	**Znchange (mg/kg)**	**Fe (mg/kg)**	**TWkg/hl**	**TKWg**	**PSI**	**Grain protein %**	W *10^**−4**^ J	**P/L**	**Loaf volume cm^**3**^**
1	8233108	VALI/3/2*QUAIU/BECARD//BECARD	7.74	106	51.7	10.7	35.6	78.9	47.3	69	12.4	230	1.4	660
2	8231520	NG8201/KAUZ/4/SHA7//PRL/VEE#6/3/FASAN/5/MILAN/KAUZ/6/ACHYUTA/7/PBW343*2/KUKUNA/8/IWA 8600211//2*PBW343*2/KUKUNA/9/KACHU*2/5/WBLL1*2/TUKURU/3/T.DICOCCON PI94624/AE.SQUARROSA (409)//BCN/4/WBLL1*2/TUKURU	7.83	106	52.7	11.7	40.2	81.3	49.0	63	12.2	165	1.1	685
3	8232992	SHAKTI/7/2*TRAP#1/BOW/3/VEE/PJN//2*TUI/4/BAV92/RAYON/5/KACHU #1/6/TOBA97/PASTOR/3/T.DICOCCON PI94624/AE.SQUARROSA (409)//BCN/4/BL1496/MILAN/3/CROC_1/AE.SQUARROSA (205)//KAUZ	7.51	105	53.9	12.9	36.8	81.1	50.6	65	13.7	308	1.1	750
4	8233528	FRET2/KUKUNA//FRET2/3/WHEAR/4/IWA 8600211//2*PBW343*2/KUKUNA/5/KACHU/BECARD//WBLL1*2/BRAMBLING/6/BOKOTA	7.62	105	49.3	8.3	42.9	80.6	52.3	66	14.0	247	1.1	765
5	8233114	VALI/3/2*QUAIU/BECARD//BECARD	7.89	104	52.2	11.2	37.0	82.5	49.9	68	12.4	245	2.9	610
6	8232346	THB/KEA//PF85487/3/DUCULA/4/WBLL1*2/TUKURU/5/IWA 8600211//2*PBW343*2/KUKUNA/6/MUTUS/AKURI #1//MUTUS/7/MUCUY	7.85	103	57.0	16.0	41.6	79.2	48.4	60	12.8	282	1.4	705
7	8233095	VALI*2/7/TRAP#1/BOW/3/VEE/PJN//2*TUI/4/BAV92/RAYON/5/KACHU #1/6/TOBA97/PASTOR/3/T.DICOCCON PI94624/AE.SQUARROSA(409)//BCN/4/BL 1496/MILAN/3/CROC_1/AE.SQUARROSA (205)//KAUZ	7.67	103	58.0	17.0	42.1	80.1	53.5	59	14.2	294	2.3	755
8	8232996	SHAKTI/7/2*TRAP#1/BOW/3/VEE/PJN//2*TUI/4/BAV92/RAYON/5/KACHU #1/6/TOBA97/PASTOR/3/T.DICOCCON PI94624/AE.SQUARROSA (409)//BCN/4/BL1496/MILAN/3/CROC_1/AE.SQUARROSA (205)//KAUZ	7.52	102	53.7	12.7	33.7	80.3	46.4	71	13.0	256	1.8	725
9	8231667	DANPHE#1*2/3/T.DICOCCON PI94625/AE.SQUARROSA (372)//SHA4/CHIL/4/WBLL1*2/KURUKU//KRONSTAD F2004/3/WBLL1*2/BRAMBLING/5/MUTUS*2/HARIL #1	8.19	102	52.0	11.0	38.1	79.6	50.7	64	12.6	299	2.3	730
10	8233575	BABAX/KS93U76//BABAX/3/ATTILA/3*BCN//TOBA97/4/WBLL1*2/KURUKU/5/IWA 8600211//2*PBW343*2/KUKUNA/6/DANPHE #1*2/SOLALA/7/SUP152/BLOUK #1	7.41	102	57.5	16.5	42.1	80.2	52.2	65	13.0	358	1.0	770
11	8233526	FRET2/KUKUNA//FRET2/3/WHEAR/4/IWA 8600211//2*PBW343*2/KUKUNA/5/KACHU/BECARD//WBLL1*2/BRAMBLING/6/BOKOTA	7.36	102	54.3	13.3	43.2	81.6	53.3	64	13.8	219	1.3	755
12	8232892	TRAP#1/BOW/3/VEE/PJN//2*TUI/4/BAV92/RAYON/5/KACHU #1/6/TOBA97/PASTOR/3/T.DICOCCON PI94624/AE.SQUARROSA (409)//BCN/4/BL 1496/MILAN/3/CROC_1/AE.SQUARROSA (205)//KAUZ/7/NADI	7.36	101	52.8	11.8	36.9	79.5	53.4	56	13.7	245	1.6	780
13	8232875	C80.1/3*BATAVIA//2*WBLL1/3/ATTILA/ 3*BCN*2//BAV92/ 4/WBLL1*2 /KURUKU/5/IWA 8600211//2*PBW343*2/KUKUNA/6 /KACHU/SAUAL/4/ATTILA*2/ PBW65//PIHA/3/ATTILA/2*PASTOR	7.13	100	53.0	12.0	33.6	79.4	49.0	67	13.1	191	1.5	670
14	8232914	KIRITATI//HUW234+LR34/PRINIA/3/GEN/OPATA/8/NG8201/KAUZ/4/SHA7//PRL/VEE#6/3/FASAN/5/MILAN/KAUZ/6/ACHYUTA/7/PBW343*2/KUKUNA/9/SUP152/BAJ #1	7.24	99	55.6	14.6	37.6	80.5	47.1	59	14.0	176	0.7	735
15	8232919	PAURAQ/4/SLM//AG/6*INIA66/3/SLM/5/PAURAQUE #1/6/BECARD #1/5/KIRITATI/4/2*SERI.1B*2/3/KAUZ*2/BOW//KAUZ	7.24	99	51.5	10.5	33.1	78.2	49.7	65	12.7	112	0.9	680
16	8231590	TRAP#1/BOW/3/VEE/PJN//2*TUI/4/BAV92/RAYON/5/KACHU #1/6/TOBA97/PASTOR/3/T.DICOCCON PI94624/AE.SQUARROSA(409)//BCN/4/BL1496/MILAN/3/CROC_1/AE.SQUARROSA (205)//KAUZ/7/FRNCLN/DANPHE	8.09	99	50.8	9.8	41.4	79.6	46.5	67	12.5	141	1.4	690
17	8231560	TRAP#1/BOW/3/VEE/PJN//2*TUI/4/BAV92/RAYON/5/KACHU #1/6/TOBA97/PASTOR/3/T.DICOCCON PI94624/AE.SQUARROSA (409)//BCN/4/BL1496/MILAN/3/CROC_1/AE.SQUARROSA (205)//KAUZ/7/MUCUY	7.25	99	60.9	19.9	48.1	79.5	57.2	52	14.4	150	0.8	755
18	8232123	PASTOR/2*SITTA//PBW343*2/KUKUNA/4/CMH80.397//RL6010/5*SKA/3/CMH80.397/5/NELOKI/7/2*TRAP#1/BOW/3/VEE/PJN//2*TUI/4/BAV92/RAYON/5/KACHU #1/6/TOBA97/PASTOR/3/T.DICOCCON PI94624/AE.SQUARROSA (409)//BCN/4/BL1496/MILAN/3/CROC_1/AE.SQUARROSA (205)//KAUZ	7.65	98	65.0	24.0	42.1	80.0	52.2	53	13.7	213	1.2	775
19	8233054	DANPHE#1*2/SOLALA//BORL14/3/BOKOTA	7.36	98	55.7	14.7	40.3	79.4	44.3	67	12.9	291	1.9	710
20	8233160	VILLA JUAREZ F2009/3/T.DICOCCON PI94625/AE.SQUARROSA (372)//3*PASTOR/4/WBLL1*2/BRAMBLING/8/PSN/BOW//SERI/3/MILAN/4/ATTILA/5/KAUZ*2/CHEN//BCN/3/MILAN/6/WBLL1*2/SHAMA/7/IWA8600211//2*PBW343*2/KUKUNA/9/SUP152/BLOUK #1	7.50	98	54.6	13.6	42.6	82.5	52.2	63	12.5	255	2.0	725

*TW, test weight; TKW, thousand kernel weight; PSI, particle size index; W, Alveograph deformation work; P/L, Alveograph ratio of dough strength and extensibility*.

## Discussion

CIMMYT's biofortification breeding program has made significant progress in developing competitive, high zinc wheat lines using landraces and wild relatives (Velu et al., 2014; Singh and Velu, 2017). The transfer of traits from wild relatives often requires considerable cytological manipulation and the incorporation of an alien chromosomal segment in the elite breeding material that will not recombine. CIMMYT has historically used translocation lines, such as 1BL.1RS (Rajaram et al., 1983; Villareal et al., 1991, 1994a). Most recently, the 2NS segment from *Ae. ventricosa* has offered novel traits, such as the *Yr17* gene for yellow rust resistance and a pleiotropic effect on wheat blast resistance.

In the 1990s, CIMMYT increased the genetic diversity of its wheat breeding program by developing synthetic wheats and crossing these with elite breeding lines (Villareal et al., 1994b). Synthetic wheats are developed by crossing the A and B genome donor (*T. dicoccum* or *T. durum*) of bread wheat with the D genome donor, *Ae. tauschii*. It is reasonable to speculate that this expansion of genetic variability may have contributed to the increase in the rate of improvement for different traits, including stress tolerance (Mondal et al., 2016), nutritional quality (Crespo-Hererra et al., 2017), industrial quality (Burnett et al., 1995) and disease resistance (Singh et al., 1998; Shumny et al., 2016).

This study describes the successful integration of novel alleles for Fe and Zn from wild relatives of wheat, by using synthetic hexaploid derivatives from tetraploid *T. dicoccum* with diploid *Ae. tauschii*. It is noteworthy that the first high Zn wheat (Zinc-Shakti = Croc_1/*Ae.squarrosa*(210)//Inqalab91^*^2/Kukuna/3/PBW343^*^2/Kukuna), which had a significantly high grain Zn concentration, was inherited from *Ae. tauschii* via synthetic wheat developed from *T. durum*× *Ae. tauschii* parents. This statement was based on the assumption that “Croc,” the (durum) parent did not have high Zn contributing alleles. In the case of “Mayil” (“WB-02”) variety, the high Zn alleles derived from both parents *T. dicoccum* and *Ae. tauschii*.

In the case of breeding for higher grain Zn, much larger segregating populations were grown to enable selection of good agronomic type and disease resistance before selecting for Fe and Zn. The resulting advanced lines were tested at CENEB, Cd. Obregon, Mexico in an Alpha lattice design with three replications yield trial, following which the high-yielding and high Zn lines were tested in second year with six artificially manipulated environments in Obregon ranging from early-sown to late-sown (heat stress) and severe drought to moderate stress by restricted irrigation systems (Velu et al., 2016) and parallel screening in multiple sites in target countries of India and Pakistan, one of the target environments for these nutrient-enriched wheat cultivars (Velu et al., 2012, 2016, 2018). Genotypes were identified that had significantly higher grain yield and grain Zn concentration across locations. In some locations, the improvement in Zn concentration was up to 30–50% higher than that of the recurrent parent. These materials represent a significant stepping stone to achieve the ultimate goal of micronutrient-enriched wheat. Competitive high Zn wheat varieties have been tested broadly for adaptation and stability in target locations and released by national programs in some developing countries (Velu et al., 2012, 2015; Baloch et al., 2015). Some of these, such as the “Zinc-Shakti,” “WB-02,” “HPBW-01,” and “Ankur Shiva” wheat varieties released in India by public and private partners and, more recently, “Nohely-F2018” released in Mexico for the Mexicali valley of northern Sonora region. Interestingly “BARI Gom 33” (= “Kachu”/“Solala”) released in Bangladesh during 2017 showed 7–8 mg/kg Zn advantage, and also offer resistance to wheat blast which is caused by *Magnaporthe oryzae*.

Another approach to increase micronutrient concentration is to use introgression segments or translocation of chromosomes from more distantly related species or unrelated species that carry the genetic code for high Fe and Zn, such as rye translocations in a Pavon wheat background. Rye and wild relatives are efficient in nutrient uptake and show adaptation in Zn deficient environments (Graham et al., 1999). In this study, some of the translocation lines showed significantly higher Zn concentration than the recurrent parent. The fact that rye is a nutrient use efficient crop suggests that some genes associated with high grain Zn and Fe might be present in the 1R chromosome (Monasterio and Graham, 2000). Genetic introgression with the short arm of rye chromosome 1 (1RS) have also generated improvements in wheat root traits (Kim et al., 2004), in addition to improved resistance to leaf rusts and powdery mildews (Villareal et al., 1994a; Ehdaie et al., 2003; Singh et al., 2011) which could contribute to better nutrient uptake. Wheat genotypes containing the rye chromosome arm 1RS are also reported to have enhanced grain yields, speculatively attributed to a superior rooting system (Villareal et al., 1991; Moreno-Sevilla et al., 1995). Bread wheat genotypes with the 1RS translocation were found to have higher root mass, thinner roots, and larger root length density in pot experiments under controlled environment conditions (Ehdaie et al., 2003). There are also reports showing differential performance for milling and processing quality (Graybosch et al., 1993; Fenn et al., 1994; Dimitrijevic et al., 2008; Zhao et al., 2012).

The development of less expensive, easier to use colorimetric assays or near infrared spectroscopy methods is essential in order to replace atomic absorption or inductively coupled plasma analysis for measuring the Zn and Fe content of the grain during breeding programs. Recently Cardoso et al. (2018) showed μ-XRF based imaging technique for localization of grain Zn and Fe in wheat. These techniques will allow breeders to test for nutrient expression in more than one environment as early as possible in the breeding process. Once advanced or pure line progeny with good levels of Fe and Zn have been identified, they must be tested across a range of locations in different years to establish the stability of nutrient expression. Significant genotype × location interaction can be expected (Ortiz-Monasterio et al., 2007; Velu et al., 2014), and the identification of genotypes with stable expression across environments is essential.

## Conclusion

About 15 and 24 percent of the breeding lines showed high yield and 12 mg/kg increase in Zn content parallelly which refers to the feasibility of developing competitive biofortified varieties with good agronomical traits and adaptability to local target environments. The positive shift in grain yield potential from 2017 to 2018 (about 0.6 t/ha) (Figure 2, Table 2) indicates great progress in terms of achieving higher genetic gain for grain yield potential of high Zn lines. Fe content was found to have high significant positive correlation with the Zn content in this experiment during 2017 (*r* = 0.46; *P* < 0.01) and 2018 (*r* = 0.50; *P* < 0.01) suggesting that simultaneous improvement of both Zn and Fe is feasible (Figure 4).

Altogether these results demonstrate that large genetic diversity is available in translocation lines for improving the nutritional content of wheat with enhanced grain Zn concentration. The wheat breeding program could effectively utilize these diverse genetic resources to improve nutritional quality of wheat, but improvement was also achieved in yield potential and wheat processing quality. Furthermore, these genetic resources are expected to improve the stress tolerance and disease resistance of the plants. Gene discovery and mapping studies would enhance breeding efficiency for high Zn content in the future.

## Author Contributions

GV conducted the field experiments and drafted the manuscript. LC, JH, RS, and TP helped with the field phenotyping and provided germplasm. CG conducted grain quality analysis.

### Conflict of Interest Statement

The authors declare that the research was conducted in the absence of any commercial or financial relationships that could be construed as a potential conflict of interest.

## Abbreviations

CV: coefficient of variation
Fe: iron
H^2^: broad sense heritability
LSD: least significant difference
PSI: particle size index
SD: standard deviation
TKW: thousand kernel weight
Zn: zinc.
